# Case of Intestinal Irritation, with Threatened Premature Labor

**Published:** 1872-12-01

**Authors:** 


### CASE OF INTESTINAL IRRITATION, WITH THREATENED PREMATURE LABOR.

BY A SENIOR STUDENT.

Oct. 1st, 5 A. M.—Was called to the bedside of Ada B——, æt. 22, a married lady, of delicate habit, whom I found *six* months in pregnancy, and laboring under a violent enteritis lit up by the indiscreet use of podophyllin, at the hands of one of those followers of that phantom-nothing, homœopathy, who had become so saturated with the idea of "attenuations" that he was actually trying to *thin* out a case of bilious remittent with doses of mandrake, directing her to take a powder as often as her bowels moved. The result can be readily imagined. I found her in the dorsal decubitus, with pulse at 130; tongue fully coated, dry and parched; urine retained; a colliquitive diarrhœa; incipient uterine contractions, with burning pain in intestines, and the abdomen swollen and distended to a degree which I had not before dreamed possible. The friends insisted that she was dying!

and she, poor woman, having "suffered many things from the physicians" (two others having preceded me), despairing of mortal succor, was looking to a higher source for help. With scarcely a shadow of hope, I asked the friends to dry their tears, and with a few words of cheer to the madam, took the case seriously in hand. Having no catheter, the bladder was emptied by application of cloths wrung out of warm water.

Fearing abortion, morphia sulph. and bismuth in iced flax-seed water was given, and continued pro. re. nat. The abdomen, anteriorly, from ensiform cartilage to line midway between umbilicus and pubis, was covered with the ceratum cantharis, and laterally with cloths wrung out of lead-water and turpentine. Drafts were applied to the feet, and half teaspoonful turpentine emulsion given every hour. At 12 M., when I left, no change was discernable. Blisters had not produced the faintest blush of irritation. I directed teaspoonful *doses* of milk and flour gruel occasionally, with an extra dose of the morphine at bed time. She rested more comfortably, but without sleep.

Oct. 2d, 9 A. M., pulse 120; tongue dry; blister had not drawn; abdomen slightly less tense; less pain on pressure; diarrhœa quite under control; uterus quiet, but with burning pain in stomach; nausea and acrid eructations, and singultus. The singultus was checked with chloroform. A mustard paste applied over stomach; suspended the turpentine emulsion and ordered the morphia and bismuth every hour, with ice, alternated with pill of nitrate of silver and opii. aa. grs. ¼. 4 P. M. Was told on arrival that mortification had set in, and patient was dying; on the contrary, found the pulse quite regular at 100; tongue moistening at tip; stomach quiet; abdomen greatly reduced in size, with little pain on pressure. Continued the pill every four hours, alternated with sol. of spts. mindererii, spts. eth. nit. syr. ipecac, and fld. ex. ver. viride. Patient rested well all night.

Oct. 3d, 9 A. M. Patient looked bright and cheerful; pulse 90; tongue moist and clearing up nicely; surface moist; fœtal movement distinctly felt by mother. Ordered v. gtts. Fowler's sol. every four hours, with Dover's powder at bed time. Patient made rapid convalescence, and without doubt will carry child to full time.

